# Clinical Manifestations, Imaging Procedures and Laboratory Parameters among Hospitalized Patients with COVID-19 in Ilam Province, Western Iran

**DOI:** 10.4314/ejhs.v32i3.3

**Published:** 2022-05

**Authors:** Mohammad Reza Kaffashian, Maryam Shirani, Maryam Koupaei, Nourkhoda Sadeghifard, Iraj Ahmadi, Aliashraf Mozafari, Ali Nazari, Mohsen Heidary, Saeed Khoshnood

**Affiliations:** 1 Department of Physiology, Faculty of Medicine, Ilam University of Medical Sciences, Ilam, Iran; 2 Student Research Committee, Ilam University of Medical Sciences, Iran; 3 Toxicology Research Center, Medical Basic Sciences Research Institute, Ahvaz Jundishapur University of Medical Sciences, Ahvaz, Iran; 4 Department of Microbiology and Immunology, School of Medicine, Kashan University of Medical Sciences, Kashan, Iran; 5 Clinical Microbiology Research Center, Ilam University of Medical Sciences, Iran; 6 Physiology Department, School of Medicine, Ilam University of Medical science, Ilam, Iran; 7 Non-Communicable Diseases Center, Ilam University of Medical Sciences, Iran; 8 Department of Infectious Disease, School of Medicine, Shahid Mostafa Khomeini Hospital, Ilam University of Medical Sciences, Ilam, Iran; 9 Department of Laboratory Sciences, School of Paramedical Sciences, Sabzevar University of Medical Sciences, Sabzevar, Iran; 10 Cellular and Molecular Research Center, Sabzevar University of Medical Sciences, Sabzevar, Iran

**Keywords:** COVID-19, Ilam, SARS-CoV-2, Iran

## Abstract

**Background:**

COVID-19 is the last global threat which WHO confirmed it as a pandemic on March 11, 2020. In the Middle East, Iran was the first country where the SARS-Cov-2 was detected. The epidemiological and economic challenges of Iran make this country a particularly relevant subject of study. In the current study, we aimed to evaluate the clinical, radiological and laboratory findings in hospitalized COVID-19 confirmed cases in Ilam province, western of Iran.

**Methods:**

Overall, 2204 hospitalized RT-PCR confirmed patients with COVID-19 were considered in this study. Electronic medical records, including clinical symptoms, radiological images, laboratory findings, and the comorbidities of patients with COVID-19 were collected and analyzed. In addition, the medication regimens used in these patients were evaluated. The patients were classified in discharged and died groups according to their outcomes. Then, clinical, radiological and laboratory findings as well as treatment regimens and underlying diseases were compared in these two groups.

**Results:**

Among the patients, 1209 (54.85%) were male and 995 (45.14%) were female. Pneumonia, dyspnea and cough, were the most common clinical data in both discharged and died groups. Among the comorbidities, COPD, and cancer were significantly more common in the dead patients than in the living. The results of laboratory tests showed that blood creatinine, BUN, ESR, Na+, WBC, and neutrophil count have increased in deceased group compared to the survivors. However, the lymphocyte count decreased in deceased patients. The evaluation of radiographs demonstrated that there were significant correlations between bilateral pneumonia, ground glass opacity, bilateral patchy shadowing, and pleural effusion with death.

**Conclusion:**

The current investigation indicated the special profile of COVID-19 in west of Iran. Discharged and dead patients with COVID-19 had distinct clinical, radiological and laboratory features, which were separated by principal component analysis. Identifying these characteristics of the disease would translate into the implementation of practical measures to improve results.

## Introduction

In December 2019, severe acute respiratory syndrome coronavirus 2 (SARS-CoV-2), the causative agent of coronavirus disease 2019 (COVID-19) infected a large number of people in Wuhan, China. This RNA virus from coronaviruses family can give rise to respiratory tract infections of different severities. SARS-CoV-2 infection varies from the normal cold to more serious conditions and is similar to the coronavirus, a main cause of SARS. SARS-CoV-2 has also been demonstrated to have a strong affinity to human respiratory receptors(1).

In recent years, global attention has been drawn to COVID-19 due to the fast-growing number of new cases, as well as its transmission via human to human interaction through droplets, contacts, and aerosols. COVID-19 has become a worldwide concern and a significant public health challenge, particularly since the rapid escalation of the infected cases and affected countries(2).

On March 11, 2020, COVID-19 was declared as a pandemic by the World Health Organization (WHO). Unlike SARS-CoV and Middle East Respiratory Syndrome (MERS)-CoV, the spread of SARS-CoV-2 is greater. In the same year, Iran was reported as one of the countries with the highest prevalence rate of cases so that it was severely hit by the severity of the virus. The first COVID-19 case recorded officially in the country (Qom province) dated on 19 February 2020(3).

The clinical presentation of mild-to-moderate COVID-19 significantly varies according to the sex and the age characteristics. Elderly patients often presented fatigue, fever, and loss of appetite, whereas young individuals more frequently had nose ear, and throat complaints (4).

Comorbidity, male sex, older age, and signs of nausea and dyspnea are estimate as higher risk factors for severe form (5–7).

In view of the fact that Iran was the first country in the Middle East where the virus was identified, it is speculated that this country is responsible for the transmission of the disease in neighboring countries, such as Iraq, Pakistan, and Afghanistan. Owing to the epidemiological conditions and the complexity of the political and economic difficulties, Iran is taken into account as a particular subject of study (8).

For the COVID-19, there are no specific clinical features but may range from no symptoms (asymptomatic cases) to severe pneumonia and death. The patients are often manifested with fever, cough, dyspnea, rhinorrhea, headache, myalgia, and arthralgia (9, 10).

Some SARS-CoV-2 infected patients also develop the severe complications of COVID-19, including acute respiratory distress syndrome (ARDS) and death; however, the reason for such behavior is unknown. Development of severe COVID-19 disease depends on multiple risk factors, comprising of sociodemographic factors and comorbid conditions (11–13).

Identification of the epidemiological, clinical and laboratory features of COVID-19 could contribute to proper decision making in the control of this epidemic disease. The present study was conducted with the aim of analyzing the epidemiological and clinical attributes of COVID-19 patients following the diagnosis of the disease by detecting the viral nucleic acid using RT-PCR.

## Material and Methods

**Study design**: We conducted the present single-center retrospective descriptive study. The study was carried out on COVID-19 cases hospitalized in Shahid Mostafa Khomeini Hospital (Ilam, Iran) and approved by the Ethics Committee of University of Medical Sciences (code of ethics: A-10-2579-5). Before initiating this research work, informed consent forms were obtained from all the patients. The approach to the disease was in accordance with the guidelines provided by Iran National Health and adapted from the WHO guidelines.

**Inclusion/exclusion criteria**: All patients who were hospitalized from 20 April 2020 to 21 May 2021 and their clinical, laboratory, and radiological information were available at the registration center were included in the study. Patients whose demographic data, laboratory tests, clinical signs, and/or radiological findings were not available in the registry system were excluded from the study. Also, pregnant patients and patients with hematological disorders were not included in the study.

**Ethical considerations**: The protocols of this study were approved by the Ethics committee of Ilam University of Medical Sciences and accomplished in conformity the ethical principles of the declaration of Helsinki. Written informed consents were received from all the patients, and their information was kept confidential.

**Clinical assessment**: Cases with fever, rhinorrhea, sore throat, cough, and probable respiratory distress were considered as patients with suspected COVID-19, particularly if they had a positive history of close relationship with a highly suspected or confirmed COVID-19 patient or had a travel history to a COVID-19-affected country or city. The disease was diagnosed considering the clinical features, chest exam, laboratory findings, and reverse-transcription polymerase chain reaction (RT-PCR) test by the use of both throat and nose swab samples.

The diagnosis of the patients was carried out clinically via lung radiographical characteristics and also verified according to the laboratory-based data, i.e., RT-PCR by throat and nose swab samples from the upper respiratory tract, a test that accurately explains the characteristics of the diagnostic kit. The extraction of total RNA and also RT-PCR for coronavirus genes were performed using High Pure RNA Isolation Kit (Roche Diagnostics, Penzberg, Germany) and Taqman® Premix (TaKaRa, Dalian, China), respectively, according to the protocol recommended by manufacturer.

**Laboratory assessment**: Peripheral venous blood samples were collected on admission or during the hospital stay. Red and white blood cell count, leukocyte subtypes count, blood type, hematocrit count, hemoglobin count, and platelet count were the routine blood tests carried out by using an automated hematology analyzer (Sysmex Corporation, Kobe, Japan). Platelet, lymphocyte, and neutrophil counts, serum urea and creatinine, C-reactive protein (CRP), erythrocyte sedimentation rate (ESR), aspartate aminotransferase (AST), alanine aminotransferase (ALT), albumin level, lactate dehydrogenase (LDH), and so on were other laboratory data.

**CT image acquisition**: Radiological evaluations were made according to CT images. Two expert radiologists assessed the presence of any radiological deformity based on the evidence or description in the medical records and finally rechecked the results. The significant CT imaging findings in each lobe, three lobes in the right lung and two lobes in the left lung, were scored. Scores of 1 and 2 were given to the alterations in Ground-glass opacities (including crazy paving) of <3 cm and >3 cm, respectively. Also, CT score of 0 was defined as normal, while those of 1-7 and 8-15 were interpreted as less than severe and severe, respectively.

**Statistical analysis**: The analysis of the data was conducted by the aid of descriptive statistics (e.g. mean, frequency tables, standard deviation, and variance) and also by analytical tests (e.g. Chi-square, Pierson correlation coefficient test, and ANOVA), using SPSS version 27. The probability level of less than 0.5 was considered to be statistically significant (*p* <0.05).

## Results

**Presenting characteristics**: The study population included 2204 patients with COVID-19 whose laboratory tests were confirmed. The mean age of patients was 56.67% who had an age range of 3-100 years. Among the patients included in this study, 451 were under the age of 40, of whom 8 died (1.8%), and 1747 were over the age of 40, of whom 94 died (5.4%). There was a significant difference between age and mortality rate. Among the patients, 1209 (54.85%) were male and 995 (45.14%) were female. In this study, 23.1% of patients were admitted to the ICU, and 3.2% of patients were rehospitalized. Among the hospitalized patients, 95.4% O_2_ demanded.

**Clinical signs and symptoms**: The most common symptoms were pneumonia (96.8%), dyspnea (87.7%), and cough (75.4%) (Table 1). Among COVID-19 comorbidities, there was a significant difference between death and the presence of chronic obstructive pulmonary disease (COPD) and cancer with *p*=0.015 and *p* =0.012, respectively. Various symptoms were reported at the onset of the disease. Among them, symptoms such as acute respiratory distress syndrome (ARDS) (*p* = 0.001), joint ache (*p* = 0.050), cough (*p* = 0.010), malaise (*p* = 0.001), nausea (*p* = 0.007), vomiting (*p* = 0.027), and myalgia (*p* = 0.001) were significantly different between the survivor and deceased groups.

**Table 1:** Demographic data and clinical features of patients with COVID-19

Variable		Total (%)	Lethal Outcome (%)	Survived (%)	p-value
**DEMOGRAPHIC DATA**					
**Age (year)**	<18	12 (0.5)	1 (1.0)	11 (0.5)	0.001
	18–59	1180 (53.7)	38 (37.2)	1142 (52.0)	
	>60	1006 (45.8)	63 (61.8)	943 (42.9)	
**Sex**	Female	995 (45.14)	40 (39.2)	953 (45.4)	0.219
	Male	1209 (54.85)	62 (60.8)	1145 (54.6)	
**PCR**	Negative	67 (3.0)	2 (2.0)	65 (3.1)	0.051
	Positive	2115 (96.0)	96 (94.1)	2019 (96.1)	
	Not defined	22 (1.0)	18 (0.9)	4 (3.9)	
**Blood Group**	O+	406 (29.1)	16 (29.3)	390 (29.3)	0.668
	A+	564 (40.5)	30 (48.4)	534 (40.1)	
	B+	227 (16.3)	6 (9.7)	221 (16.6)	
	AB+	91 (6.5)	5 (8.1)	86 (6.5)	
	A-	25 (1.8)	2 (3.2)	23 (1.7)	
	B-	20 (1.4)	1 (1.6)	19 (1.4)	
	AB-	9 (0.6)	0 (0.0)	9 (0.7)	
	O-	52 (3.7)	2 (3.2)	50 (3.8)	
**MANIFESTATIONS**					
**Dyspnea**	Yes	1932 (87.7)	93 (91.2)	1839 (87.5)	0.206
	No	272 (12.3)	9 (8.8)	263 (12.5)	
**Discharge from the hospital**	Yes	1864 (96.6)	3 (8.8)	1861 (98.2)	0.001
	No	66 (3.4)	31 (91.2)	35 (1.8)	
**Pneumonia**	Yes	1818 (96.8)	90 (94.7)	1728 (96.9)	0.226
	No	60 (3.2)	5 (5.3)	55 (3.1)	
**Cough**	Yes	1662 (75.4)	66 (64.7)	1596 (75.9)	0.010
	No	542 (24.6)	36 (35.3)	506 (24.1)	
**Myalgia**	Yes	1194 (54.2)	38 (37.3)	1156 (55.1)	0.001
	No	1007 (45.8)	64 (62.7)	943 (44.9)	
**Fever**	Yes	1182 (53.6)	52 (51.0)	1130 (53.8)	0.583
	No	1022 (46.4)	50 (49.0)	972 (46.2)	
**Malaise**	Yes	770 (35.0)	52 (51.9)	718 (34.2)	0.001
	No	1433 (65.0)	49 (48.5)	1384 (65.8)	
**ARDS**	Yes	175 (7.9)	28 (27.5)	147 (7.0)	0.001
	No	2029 (92.1)	74 (72.5)	1955 (93.0)	
**Nausea**	Yes	651 (29.5)	18 (17.6)	633 (30.1)	0.007
	No	1553 (70.5)	84 (82.4)	1469 (69.9)	
**Headache**	Yes	566 (25.7)	22 (21.6)	544 (25.9)	0.330
	No	1638 (74.3)	80 (78.4)	1558 (74.1)	
**Vomiting**	Yes	499 (22.7)	14 (13.7)	485 (23.1)	0.027
	No	1702 (77.3)	88 (86.3)	1614 (76.9)	
**Loss smell**	Yes	307 (14.1)	12 (12.9)	295 (14.2)	0.733
	No	1869 (85.9)	81 (87.1)	1788 (85.8)	
**Loss taste**	Yes	305 (14.0)	12 (13.2)	293 (14.0)	0.817
	No	1872 (86.0)	79 (86.8)	1793 (86.0)	
**Diarrhea**	Yes	193 (8.8)	7 (6.9)	186 (8.8)	0.488
	No	2011 (91.2)	95 (93.1)	1916 (91.2)	

**Radiological findings**: The results of CT scan and radiographs of patients' lungs showed different patterns (Table 2).

**Table 2:** Radiological findings and comorbidities of patients with COVID-19

Variable		Total (%)	Outcome		p-value
				
			Lethal (%)	Survived (%)	
**COMORBIDITIES**					
**Diabetes**	Yes	501 (22.8)	25 (24.8)	476 (22.7)	0.623
	No	1701 (77.2)	76 (75.2)	1625 (77.3)	
**Heart disease**	Yes	482 (21.9)	25 (24.5)	457 (21.8)	0.517
	No	1717 (78.1)	77 (75.5)	1640 (78.2)	
**COPD**	Yes	120 (5.4)	11 (10.8)	109 (5.2)	0.015
	No	2084 (94.6)	91 (89.2)	1993 (94.8)	
**Kidney disease**	Yes	100 (4.5)	7 (6.9)	93 (4.4)	0.224
	No	2104 (95.5)	95 (93.1)	2009 (95.6)	
**Neurologic disease**	Yes	99 (4.5)	5 (4.9)	94 (4.5)	0.805
	No	2104 (95.5)	97 (95.1)	2007 (95.5)	
**Metabolic disease**	Yes	69 (3.1)	1 (1.0)	68 (3.2)	0.373
	No	2134 (96.9)	101 (99.1)	2033 (96.8)	
**Smoking**	Smoke	62 (2.8)	5 (4.9)	57 (2.7)	0.290
	Ex-smoke	20 (0.9)	2 (2.0)	18 (0.9)	
	No	2122 (96.0)	95 (93.1)	2027 (96.4)	
**Dialysis**	Yes	61 (2.8)	4 (3.9)	57 (2.7)	0.527
	No	2143 (97.2)	98 (96.1)	2045 (97.3)	
**Immunodeficiency**	Yes	60 (2.7)	3 (2.9)	57 (2.7)	0.756
	No	2143 (97.3)	99 (97.1)	2044 (97.3)	
**Thyroid disorders**	Yes	52 (2.4)	0 (0.0)	52 (2.5)	0.083
	No	2152 (97.6)	102 (100)	2050 (97.5)	
**Psycho disease**	Yes	44 (2.0)	1 (1.0)	43 (2.0)	0.720
	No	2159 (98.0)	100 (99.0)	2059 (98.0)	
**Addiction**	Yes	41 (1.9)	2 (2.0)	39 (1.9)	0.715
	No	2163 (98.1)	100 (98.0)	2063 (98.1)	
**Liver disease**	Yes	22 (1.0)	1 (1.0)	21 (1.0)	1.000
	No	2182 (99.0)	102 (99.1)	2081 (99.0)	
**Pregnancy**	Yes	13 (0.6)	0 (0.0)	13 (0.6)	1.000
	No	2191 (99.4)	102 (100.0)	2089 (99.4)	
**RADIOLOGY DATA**					
**Multiple mottling and** **ground glass opacity**	Yes	964 (43.7)	60 (6.2)	904 (6.2)	0.002
	No	1240 (56.3)	42 (3.4)	1198 (96.6)	
**Bilateral pneumonia**	Yes	799 (36.3)	56 (54.9)	743 (93.0)	0.001
	No	1405 (63.7)	46 (3.3)	1359 (96.7)	
**Consolidation**	Yes	209 (9.5)	14 (6.7)	195 (93.3)	0.163
	No	1995 (90.5)	88 (4.4)	1907 (95.6)	
**Peripheral distribution**	Yes	184 (8.3)	6 (3.3)	178 (96.7)	0.463
	No	2020 (91.7)	96 (4.8)	1924 (95.2)	
**Bilateral patchy shadowing**	Yes	152 (6.9)	14 (9.2)	138 (90.8)	0.014
	No	2052 (93.1)	88 (4.3)	1964 (95.7)	
**Pleural effusion**	Yes	70 (3.2)	9 (12.9)	61 (87.1)	0.004
	No	2134 (96.8)	93 (4.4)	2041 (95.6)	
**Crazy paving**	Yes	26 (1.2)	3 (11.5)	23 (88.5)	0.116
	No	2178 (98.8)	99 (4.5)	2079 (95.5)	

**Figure 1:**
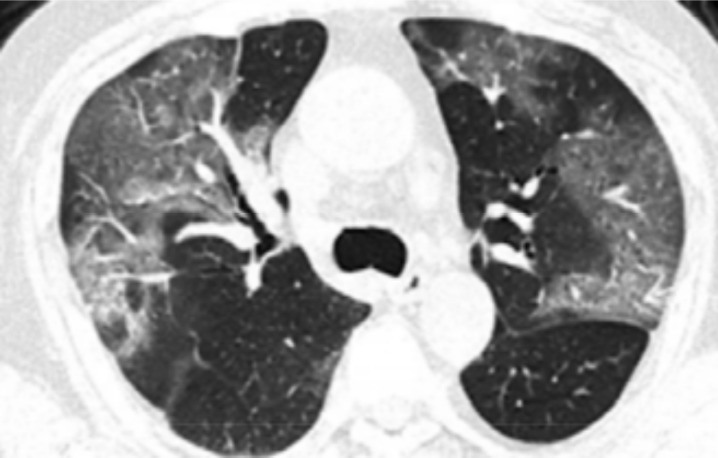
COVID-19 pneumonia: section CT shows bilateral GGO

**Figure 2:**
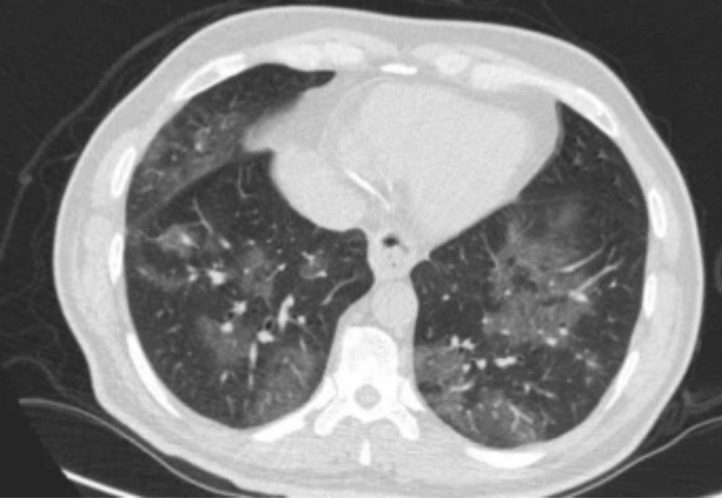
COVID-19 pneumonia: section CT shows bilateral multifocal subpleural and GGO

The most common abnormality was multiple mottling and ground glass opacity (964: 43.7%) (Figure 1), followed by bilateral pneumonia (799: 36.3%) (Figure 2). No cases of cavitation were observed. There were significant correlations between bilateral pneumonia, multiple mottling and ground glass opacity, bilateral patchy shadowing, and pleural effusion with death. There was a significant difference between the number of lung lobes involved in the disease and the incidence of death.

**Laboratory findings**: According to the observations, blood creatinine levels have increased in deceased group compared to the survivors, which based on statistical analysis is a significant. In deceased patients, white blood cell (WBC) (*p* = 0.001) and absolutely neutrophil count (*p* = 0.001) increased and absolutely lymphocyte count (*p* = 0.001) decreased. Calcium and sodium levels were decreasing and increasing in the deceased group, respectively. Although no significant correlation was reported with calcium depletion, this correlation was significant for sodium increment. Alkaline phosphatase (ALP), albumin, carbon dioxide and ferritin decreased and bilirubin, aspartate aminotransferase (AST), blood urea nitrogen (BUN), glucose, Lactate dehydrogenase (LDH), alanine aminotransferase (ALT) and Creatine Phosphokinase (CPK) increased in the deceased group but no significant correlation was observed. There was a significant increase in erythrocyte sedimentation rate (ESR) in the deceased group with *p* = 0.001. Although C-reactive protein (CRP) was increased in the deceased group, there was no significant difference between CRP and mortality rates. Information on laboratory findings is available in Table 3.

**Table 3:** Laboratory findings of patients with COVID-19

Findings	Mean Total (±SD) (n= 2204)	Mean (±SD)		p-value
		Lethal Outcome (n= 308)	Survived (n= 1896)	
**Total protein**	6.16 (±1.458)	5.80 (±0.458)	6.20 (±1.535)	0.662
**Creatinine**	1.37 (±1.523)	1.79 (±1.375)	1.35 (±1.527)	0.003
**RBC**	8.35 (±161.412)	9.32 (±48.043)	8.30 (±164.867)	0.951
**WBC**	8618.79 (±9279.467)	12082.83 (±7360.679)	8453.44 (±9330.544)	0.001
**Hematocrit**	39.78 (±6.267)	39.10 (±6.845)	39.82 (±6.238)	0.265
**Hemoglobin**	13.43 (±2.347)	13.06 (±2.260)	13.45 (±2.350)	0.112
**PLT**	212928.82 (±98103.822)	208846.94 (±82438.650)	213122.72 (±98799.001)	0.673
**Lymphocyte**	22.52 (±11.184)	17.40 (±11.913)	22.77 (±11.092)	0.001
**Neutrophil**	75.81 (±11.932)	81.60 (±12.642)	75.54 (±11.830)	0.001
**Calcium**	14.91 (±231.746)	10.31 (±9.171)	15.13 (±237.241)	0.844
**Phosphorus**	3.46 (±0.690)	3.77 (±1.576)	3.45 (±0.621)	0.155
**Magnesium**	2.16 (±0.524)	2.21 (±0.399)	2.16 (±0.529)	0.428
**Sodium**	137.92 (±6.623)	139.42 (±4.595)	137.85 (±6.695)	0.024
**Potassium**	4.15 (±4.442)	4.55 (±3.804)	4.13 (±4.469)	0.380
**BUN**	41.91 (±33.351)	64.27 (±49.968)	40.84 (±31.971)	0.001
**Total bilirubin**	1.00 (±5.203)	117.7273 (±60.22639)	0.91 (±4.801)	0.084
**AST**	52.09 (±96.446)	67.91 (±93.271)	51.38 (±96.550)	0.136
**ALT**	42.61 (±71.838)	54.54 (±79.754)	42.10 (±71.457)	0.142
**Albumin**	5.42 (±34.010)	3.74 (±0.700)	5.51 (±34.898)	0.697
**Glucose**	145.31 (±92.129)	159.36 (±97.256)	144.66 (±91.860)	0.146
**LDH**	781.09 (±6637.455)	907.39 (±887.942)	774.93 (±6794.664)	0.865
**CPK**	235.10 (±475.114)	416.00 (±1224.209)	223.35 (±377.245)	0.233
**CRP**	1.83 (±1.210)	1.73 (±1.187)	1.83 (±1.211)	0.470
**ESR**	37.84 (±28.461)	48.27 (±30.392)	37.38 (±28.292)	0.001
**D-dimer**	0.2037 (±0.30444)	0.3462 (±0.32046)	0.1986 (±0.30309)	0.086
**Fever degree**	37.98 (±1.186)	37.28 (±5.212)	38.01 (±0.491)	0.363

**Abbreviations**: F: female, M: male, ESR: erythrocyte sedimentation rate, AST: aspartate aminotransferase, ALT: alanine aminotransferase, LDH: lactate dehydrogenase, BUN: Blood urea nitrogen, PT: prothrombin time, PTT: partial thromboplastin time, CPK: Creatine phosphokinase, WBC: White Blood Cell, RBC: Red Blood Cell

**Interventions**: According to Iranian treatment protocols, two groups of drugs were used to treat COVID-19. The first group included oseltamivir, hydroxychloroquine, ribavirin and lopinavir/ritonavir and the second group included recigen, zifron, vit D and remdesivir (Table 4). Among the various drug regimens, the most common was the combination of oseltamivir, hydroxychloroquine, and lopinavir/ritonavir (45.5%). There was a significant difference between survivor and deceased groups receiving plasma therapy (*p* = 0.049). During treatment, 96.1% of patients needed oxygen so they were intubated or used mechanical intubation. There was a significant difference between the two groups in both intubation (*p* = 0.001) and mechanical intubation (*p* = 0.001) methods (Table 4).

**Table 4:** Medication regimens and treatments used in patients with COVID-19

Medication regimens and treatments		Total (%) No (%)	Lethal Outcome No (%)	Survived No (%)	p-value
**OST + HCQ + LPV/r**	Yes	1007 (45.7)	38 (37.2)	969 (46.1)	0.056
	No	1202 (54.3)	65 (62.8)	1137 (53.9)	
**Intubation**	Yes	303 (13.7)	89 (87.3)	214 (10.2)	0.001
	No	1901 (86.3)	13 (12.7)	1888 (89.8)	
**Mechanical intubation**	Yes	264 (12.0)	73 (71.6)	191 (9.1)	0.001
	No	1940 (88.0)	29 (28.4)	1911 (90.9)	
**Recigen + Vit D + Remdesivir**	Yes	259 (11.8)	7 (6.9)	252 (12.0)	0.116
	No	1945 (88.2)	95 (93.1)	1850 (88.0)	
**Recigen + Vit D**	Yes	109 (4.9)	1 (1.0)	108 (5.1)	0.059
	No	2095 (95.1)	101 (99.0)	1994 (94.9)	
**OST + HCQ**	Yes	93 (4.2)	6 (5.9)	87 (4.1)	0.442
	No	2111 (95.8)	96 (94.1)	2015 (95.9)	
**Plasma therapy**	Yes	58 (2.6)	6 (5.9)	52 (2.5)	0.049
	No	2146 (97.4)	96 (94.1)	2050 (97.5)	
**Recigen or Zifron**	Yes	27 (1.2)	0 (0.0)	27 (1.0)	0.634
	No	2177 (98.8)	102 (100.0)	2075 (98.7)	
**OST + HCQ + LPV/r + Ribavirin**	Yes	6 (0.3)	1 (1.0)	5 (0.2)	0.248
	No	2198 (99.7)	101 (99.0)	2097 (99.8)	
**OST + HCQ**		93 (4.3)	6 (6.9)	87 (4.1)	
**OST + HCQ + LPV/r**		1007 (45.7)	38 (37.2)	969 (46.1)	
**None**		1102 (50.0)	57 (55.9)	1045 (49.8)	
**Recigen + Zifron + Vit D**		103 (4.7)	1 (1.0)	102 (4.8)	
**Recigen + Zifron + Vit D + Remdesivir**		275 (12.5)	7 (6.9)	268 (12.7)	
**None**		1800 (82.8)	94 (92.1)	1706 (82.5)	

**Abbreviations**: OST: oseltamivir, HCQ: hydroxychloroquine, LPV/r: lopinavir / ritonavir, Vit: vitamin

**Outcomes**: The mortality rate among patients was 14%, of which 4.6% of deaths were due to COVID-19. Most causes of death for reasons other than COVID-19 included diabetes, high blood pressure, heart attack, and heart failure. The results showed that history of contact with suspected cases (*p* = 0.001) and history of contact with dyspnea cases had significant difference (*p* = 0.001). Patients with a history of contact with suspected and dyspnea cases had a higher mortality rate. The average number of admission days in ICU in the survivor and deceased groups was 4.79 (±5.292) and 6.43 (±5.961), respectively.

There was a significant difference between the number of hospitalization days and death (*p* = 0.024). Among the patients studied in this study, 1864 (96.6%) were discharged from the hospital. Analyzes showed that there was a significant difference and an inverse correlation between partial recovery with death rate in patients. Patients who had a history of visits medical centers two weeks before hospitalization were more likely to die than those without a history, which showed a significant difference (*p* = 0.001). Also, the analyses showed a significant correlation between the history of visiting medical centers and the rate of mortality.

## Discussion

This retrospective study reports the demographics, clinical symptoms, and the results of laboratory tests findings of 2204 patients with confirmed COVID-19 infected, who were treated at Shahid Mostafa Khomeini Hospital, (Ilam, Iran).

Although the number of infected men was more than women, this difference was not significant. In a meta-analysis study, Packham et al reported that there was no difference in the proportion of men and women with COVID-19 (14). However, Bwire reported that biological and lifestyle differences have led to reports in various studies that men are more likely to be infected than women. Of course, women are more likely than men to take preventive measures, such as the use of face masks and frequent hand washing (15).

The average age of the patients was 56.67 years but most deaths occur at an average age of 64.16 years. Mortality was significantly higher in people over 40 years of age, which is almost in the age range reported by the study of Nikpouraghdam et al in Iran (16). Based on these results, old age can be considered as a risk factor for death.

The frequency of blood groups A^+/-^, O^+/-^, B^+/-^ and AB^+/-^ among patients was estimated at 40.5/1.4%, 29.1/3.7%, 16.3/1.4% and 6.5/0.6% respectively. The frequency of blood groups A^+/-^, O^+/-^, B^+/-^ and AB^+/-^ among individuals who died of this infection was estimated at 48.4/3.2%, 29.3/3.2%, 9.7/1.6% and 8.1/0.0% respectively. This study confirmed the relationship between ABO blood groups and COVID-19 sensitivity in patients. Patients with blood type A had a higher frequency compared to non-blood type A and patients with blood type AB had a much lower frequency compared to non-blood type AB.

In the meta-analysis performed by Liu, blood groups A and B were significantly more at risk for COVID-19, whereas this was not the case for blood group AB, people with blood type O were not susceptible to the disease (17). The researchers found that in people with blood type O, the production of natural anti-A and anti-B antibodies could potentially prevent viral attachment to host cells, a mechanism that could explain their lower risk of infection compared to other blood groups (18).

However, in this study, blood group O along with blood group A are more common among patients, which may be due to the fact that blood group O (36.49%) and A (32.09%) are the most blood common group among Iranians (19).

The most common symptoms in patients referred to the hospital were pneumonia (96.8%), dyspnea (87.7%); cough (75.4%); myalgia (54.2%); fever (53.6%); and shiver (48.7%). The proportion of patients who developed dyspnea in our analysis (87.7%) was more than that reported from meta-analysis done in China and other countries, where over 33.9% of the patients examined had dyspnea (20). As stated in other studies on the clinical signs of COVID-19, few patients had prominent upper respiratory tract signs and symptoms (runny nose (5.9%) or sore throat (19.5%)), indicating that the target cells might be located in the lower airway, furthermore, COVID-19 patients rarely developed gastrointestinal signs and symptoms (diarrhea (8.8%), eg) (12).

In this study, patients with severe illness developed ARDS (7.9%), required ICU admission (23.1%), intubation (13.7%), mechanical intubation (13.7) and oxygen therapy (96.1%). Among those who died, 99% needed oxygen, 89% needed incubation, and 71% needed mechanical incubation. The need for invasive mechanical ventilation in this patient population was less than that in Italy (88%) (21), and equal to Washington State (71%) (22). The mortality rate in patients who required ICU and mechanical intubation was statistically significantly higher than patients who did not require ICU and mechanical intubation.

According to our results, a total of 54.7% of patients had at least one underling disease in line with that reported by Grasselli et al (68%)(21). As with other studies (16, 23), our results it also showed that having co-morbidities can have a statistically significant effect on mortality. Compared to the two groups, only the presence of cancer and COPD was statistically significant.

Based on our data, most abnormal radiologic findings consisted of bilateral pneumonia, multiple mottling and ground-glass opacity, bilateral patchy shadowing, and pleural effusion. As with other publications, our data show that CT scans can play an important role in diagnosing and assessing the severity of the disease (24). Jarineshin et al have referred to bilateral pneumonia and bilateral ground-glass opacities on CT scans of people with COVID-19, which have also been seen in our study (25).

These symptoms were more common in the deceased patients than in the survivors.

Among laboratory findings, WBC, absolute neutrophil count, Na+, BUN and ESR were higher among in the deceased patients in comparison to the survivors, and the results are in accordance with the previous studies (26). According to the results of a meta-analysis study, patients with COVID-19 with lymphopenia are more likely to develop severe disease (27). In the present study, the number of lymphocytes decreased in people who died compared to those who survived. Contact with infected people has played an important role in the spread of the disease, according to another study(28).

In this study, the history of occupational contact, contact with suspected cases and referral to medical centers during the two weeks before hospitalization was significantly higher in people who died than in other patients. In Brazil, a total of 34.4% patients had a recent international travel history and 61.1% patients had a history of close contact either with a positive or suspected case of COVID-19 (29). In the study of Qiao et al 53.33% of patients had the history of travel to Wuhan, 26.67% of patients had close contact with confirmed patients, and 6.67% of patient had close contact with suspected patients (30).

In the study of Nopsopon et al no participants with a history of travel to the high-risk area or close contact with PCR-confirmed COVID-19 case developed SARS-CoV-2 antibodies. No association between history of travel to a high-risk area and close contact with PCR-confirmed or suspected COVID-19 case, was found (31).

Some people believe that alcohol consumption is beneficial for the prevention and treatment of COVID-19 (32). Among patients, 11 (0.5%) patients consumed alcohol and 82 (3.70%) were current smokers; also 41 (1.9%) patients were addicted. No relationship was found between severity of COVID-19 and smoking, and drinking alcohol in this study. The prevalence of low alcohol consumption in our study is probably due to the fact that in Iran, like many Muslim majority countries where alcohol consumption is prohibited. Dai's findings indicated that COVID-19 patients with a history of cigarette smoking tend to have more severe outcomes than non-smoking patients. However, alcohol consumption did not reveal significant effects on neither development of severe illness nor death rates in COVID-19 patients (33).

In the study of Saurabh et al, alcohol use was found to increase the risk of symptomatic disease as compared with asymptomatic infection. Current tobacco smoking but not smokeless tobacco use appeared to reduce the risk of symptomatic disease (34). The smoking and drinking chewing rates in Zhong's study was 15.4 and 26.4%, respectively. The chi-square test showed no statistical significance with the classification of COVID-19. The smoking rate of COVID-19 patients was lower than that the general population (35).

Two groups of drugs were used to manage COVID-19 based on Iranian treatment protocols and disease severity in individual. Group one included oseltamivir, hydroxychloroquine, Ribavirin and lopinavir/ritonavir. There was no significant difference between the group of survivors and the deceased patients used this drug group. A clinical trial was conducted in United Kingdom, to investigate various drug candidates or therapies including hydroxychloroquine against severe COVID-19. The result demonstrated no efficacy of hydroxychloroquine against COVID-19 (36). In this study, there was no significant difference between the group of survivors and the deceased patients used hydroxychloroquine and oseltamivir. But in the second group, which included recigen and zifron as interferon-β1b, vit D and remdesivir, there was a significant difference between the survivors and the deceased patients.

Zhaori et al, found that among the reports on monotherapies, only remdesivir, and among combined antiviral agents, only the combined regimen with interferon-β1b, lopinavir-ritonavir and ribavirin were effective and safe based on evidences from RCTs (37). In the study of Pan et al, remdesivir, hydroxychloroquine, lopinavir, and interferon regimens had little or no effect on hospitalized patients with COVID-19, as indicated by overall mortality, initiation of ventilation, and duration of hospital stay (38).

Data from Beigel et al show that remdesivir was superior to placebo in shortening the time to recovery in adults who were hospitalized with COVID-19 and had evidence of lower respiratory tract infection (39). Hensley et al reported that IFN-β-1a could be an effective therapeutic agent for SARS-CoV infections. In that study, IFN-β-1a demonstrated potent antiviral activity and acceptable safety profiles, suggesting its efficacy in coronavirus treatment (40).

These data suggest that mortality was associated with older age, multiple co-morbidities, abnormal CT scans at admission, direct admission to the ICU, low lymphocyte count, history of suspected exposure, and intubation. Also, drugs including interferon beta (recigen or zifron) and remdesivir are also effective in reducing mortality.

There are several limitations to this study. First, as our study was restricted to a single center in Ilam province, it is better to interpret it more carefully for other centers. Second, PCR-positive patients were enrolled in the study. Thus, the existence of publication bias should be considered. Third, it should be considered that false-negative and false-positive PCR may occur during the detection of SARS-CoV-2 virus. This issue affects the cases studied.

In conclusion, according to the results of the current study, it can be concluded mortality was associated with older age, multiple co-morbidities, abnormal CT scans at admission, direct admission to the ICU, low lymphocyte count, history of suspected exposure, and intubation. In fact, it seems that COVID-19 patients in west of Iran have a special profile of disease. Identifying the characteristics of the disease would translate into the implementation of practical measures to improve results.
